# Control of pyrethroid-resistant *Anopheles gambiae* s.l. with Sovrenta® 15WP, a new isoxazoline insecticide for indoor residual spraying

**DOI:** 10.1186/s12936-026-05810-6

**Published:** 2026-02-27

**Authors:** Renaud Govoetchan, Abel Agbevo, Juniace Ahoga, Hospice Avanon, Thomas Syme, Boris N’dombidge, Victoria Ariori, Damien Todjinou, Laurette Kiki, Corine Ngufor

**Affiliations:** 1 London School of Hygiene and Tropical Medicine (LSHTM), Liverpool School of Tropcal Medicine, London, WC1E 7HT UK; 2 Centre de Recherches Entomologiques de Cotonou (CREC), Liverpool School of Tropcal Medicine, Cotonou, Benin; 3 Panafrican Malaria Vector Research Consortium (PAMVERC), Liverpool School of Tropcal Medicine, Cotonou, Benin; 4 African Institute for Research in Infectious Diseases (AIRID), Liverpool School of Tropcal Medicine, Cotonou, Benin

**Keywords:** Isoxazoline, Isocycloseram, Indoor residual spraying, Malaria vectors, Sovrenta, Actellic, Cove Benin

## Abstract

**Introduction:**

An expanded portfolio of more effective WHO-prequalified insecticides for indoor residual sparing (IRS) is needed to provide additional options to disease control programmes and enhance their capacity to efficiently apply IRS rotations for managing vector resistance to insecticides. We investigated the efficacy and residual activity of Sovrenta® 15WP, a wettable powder formulation of the newly discovered isoxazoline insecticide isocycloseram, (active ingredient trademarked as PLINAZOLIN® technology) for IRS in laboratory bioassays and experimental hut studies.

**Methods:**

Sovrenta® 15WP, was evaluated under laboratory conditions for 12 months at the dose of 120 mg a.i./m^2^ on cement, mud and wood block substrates against insecticide-susceptible *Anopheles gambiae* sensu stricto Kisumu and pyrethroid-resistant *An. gambiae* sensu lato (s.l.) Covè strains. An experimental hut trial was also performed to investigate its efficacy and residual activity on cement and mud-plastered walls at the target dose of 120 mg a.i./m^2^ over 12 months against wild free-flying pyrethroid-resistant *An. gambiae* sl at the Covè experimental hut station in Benin. Mosquito mortality was recorded every 24 h for up to 168 h post-exposure. Sovrenta® 15 WP was compared to Actellic® 300CS, a WHO/PQ-listed pirimiphos-methyl IRS insecticide applied at 1000 mg a.i./m^2^

**Results:**

In laboratory cone bioassays, Sovrenta® 15WP induced > 80% mortality of susceptible and pyrethroid-resistant *An. gambiae* sl for 11–12 months on cement, mud and wood block substrates. A total of 12,850 wild pyrethroid-resistant *An. gambiae* s.l. were collected in the experimental hut trial. Sovrenta® 15WP induced significantly higher mosquito mortality in the experimental huts over 12 months compared to Actellic® 300CS (68–72% vs 44–46%, *p* < 0.001). The insecticide also demonstrated a delayed mortality effect against wild vector mosquitoes that increased gradually from 25 to 42% at 24 h to 68–72% at 168 h post-exposure. Vector mortality did not differ substantially between the different substrate types. The odds ratio describing the difference in overall mortality between Actellic® 300CS and Sovrenta® 15WP was 3.38 (95% CI: 2.90–3.94) in cement-walled huts, 2.49 (95% CI: 2.10–2.95) in mud-walled huts and 1.80 (95% CI:1.53–2.11) when data for both hut wall substrate types was combined. Using recent WHO guidelines for determining non-inferiority, Sovrenta® 15WP was non-inferior and superior to Actellic® 300CS for the primary end-point of mosquito mortality over the 12-month experimental hut trial. Mortality in in situ hut wall cone bioassays was > 80% for 12 months with Sovrenta® 15WP and 9 months with Actellic® 300CS.

**Conclusion:**

Sovrenta® 15WP provided extended control of pyrethroid-resistant malaria vectors when applied for IRS on local wall substrates. The insecticide presents a new effective IRS option for achieving improved malaria control and managing insecticide resistance through the rotation of IRS insecticides.

## Background

Indoor residual spraying (IRS) is the application of long-lasting insecticide to the walls and other potential resting surfaces of disease vectors in human dwellings, targeting them to eliminate or reduce their ability to transmit diseases to humans. IRS is recognized as a core intervention for malaria prevention and control [1]. Historically, it played a key role in eliminating malaria from 37 countries during the Global Malaria Eradication Campaign (1955–1969) [2] and, more recently, contributed to significant reductions in malaria burden between 2000 and 2015 [3, 4]. However, the impact of IRS for malaria control is increasingly threatened by the development of vector resistance to insecticides, as well as declining coverage due to the high costs and logistical difficulties associated with implementation [5, 6]. In response to these challenges, new insecticides with novel modes of action that can provide improved and prolonged control of malaria vector populations that have developed resistance to existing products, are being developed for IRS. These insecticides can be deployed in high-endemic settings with extended transmission seasons to achieve sustained malaria control. They also improve capacity to manage insecticide resistance through the IRS rotation strategy recommended by the World Health Organization’s (WHO) Global Plan for Insecticide Resistance Management [7].

In the past decade, the World Health Organization has prequalified IRS insecticide formulations containing two new modes of action; the neonicotinoid clothianidin and, more recently, the meta-diamide broflanilide [8]. Both types of IRS insecticides have shown improved and prolonged efficacy against malaria vectors in various settings [9–13]. However, a more diverse portfolio of WHO-prequalified IRS insecticides is still needed to provide disease control programs with additional options and to strengthen their capacity to implement IRS rotations effectively for managing insecticide resistance in malaria vectors [13].

Sovrenta® 15WP is a wettable powder formulation of isocycloseram, (active ingredient trademarked as PLINAZOLIN® technology) a newly discovered insecticide by Syngenta Crop Protection AG. The active ingredient is a novel isoxazoline insecticide and acaricide with activity against lepidopteran, hemipteran, coleopteran, thysanopteran and dipteran pest species [14]. It selectively targets the invertebrate Rdl GABA receptor at a site that is distinct to fiproles and organochlorine sites within insects [15–17]. Detailed studies demonstrated that the binding sites relevant to the insecticidal activity of avermectins and isocycloseram are also distinct [14]. The widely distributed cyclodiene resistance mutation, A301S, does not affect sensitivity to isocycloseram, either in vitro or in vivo, demonstrating the suitability of *isocylsoseram* to control pest infestations with this resistance mechanism. The isoxazoline isocycloseram has been classified in Group 30 “GABA-Gated Chloride Channel Allosteric Modulators” of the Insecticides Resistance Action Committee (IRAC) [14].

To be prequalified by WHO and considered for large-scale deployment by disease control programmes, new IRS insecticides must undergo rigorous laboratory and semi-field experimental hut studies to demonstrate their efficacy against target disease vectors. In this study, we evaluated the efficacy and residual activity of Sovrenta® 15WP for IRS on various local wall substrates, testing its performance against both laboratory-maintained susceptible and pyrethroid-resistant malaria vectors through a series of laboratory bioassays. Additionally, we evaluated its efficacy against wild, free-flying pyrethroid-resistant *Anopheles gambiae* sensu lato in experimental huts in Covè, southern Benin. Sovrenta® 15WP was compared for non-inferiority to Actellic® 300CS, a WHO-prequalified organophosphate IRS insecticide. The study was conducted in accordance with OECD Good Laboratory Practice (GLP) principles at the CREC-LSHTM GLP-certified facility in Benin. Experimental procedures followed current WHO guidelines for evaluating IRS insecticides [18].

## Materials and methods

### Laboratory evaluation of Sovrenta® 15WP

#### Preparation and treatment of block substrates

Sovrenta® 15WP was evaluated under laboratory conditions on cement, mud, and wood block substrates. Blocks were moulded in petri dishes (9 cm diameter, 1 cm thickness) and dried at 30 ± 2 °C and 80 ± 10% relative humidity for 30 days before insecticide application. Cement blocks were made using a 1:1 mixture of cement and sand, while mud blocks were prepared from local mud paste with 10% cement added to improve durability and reduce cracking, following local construction practices. Wood blocks were cut from planks commonly used for house construction in Benin. Four replicate blocks were prepared for each treatment arm. Before IRS application, the potential of hydrogen (pH) of each block type was recorded. Treatments were applied using a Potter spray tower (Burkard Manufacturing Co Ltd) as recommended by WHO, ensuring a precise and uniform application of the target concentration of active ingredient (a.i.) per unit area [19]. Treated blocks were weighed before and after application to verify the amount of insecticide applied.

#### Residual efficacy of Sovrenta® 15WP on block substrates

Following a series of preliminary laboratory studies, an application rate of 120 mg a.i./m^2^ was identified as a suitable field dose for Sovrenta® 15WP. The insecticide also demonstrated a delayed mortality effect against mosquitoes, lasting up to 7 days post-application. To evaluate its residual efficacy on cement, mud, and wood substrates under laboratory conditions, the block substrates were treated at the specified application rate and tested in WHO cone bioassays 1 week after treatment and then monthly for up to 12 months, using both susceptible and pyrethroid-resistant strains of *Anopheles gambiae*. For each mosquito strain and substrate type, 40 unfed female mosquitoes (2–5 days old) were exposed to treated surfaces in cone bioassays for 30 min, in cohorts of 10 mosquitoes per block, following WHO guidelines [19]. After exposure, mosquitoes were held under standard conditions of 27 ± 2 °C and 80 ± 10% relative humidity. Knockdown was recorded after 1 h, and delayed mortality was assessed every 24 h for up to 168 h.

#### Mosquito strains tested in laboratory bioassays

The cone bioassays were performed with the following strains maintained at CREC/LSHTM insectary in Cotonou, Benin.

- *An. gambiae* sensu stricto Kisumu, an insecticide-susceptible reference strain originating from the Kisumu area in Kenya was colonized at the CREC/LSHTM insectary.
- *An. gambiae* sensu lato (s.l.) Covè strain is an insecticide-resistant field strain which are F1 progeny of mosquitoes collected from the CREC/LSHTM field station in Covè (7° 14′ N2° 18′ E), southern Benin. The strain exhibits a high frequency of resistance to pyrethroids and organochlorines but remains susceptible to other insecticide classes [20]. The strain is composed of a mixture of *An. coluzzii* and *An. gambiae* s.s.. Resistance is mediated by a target site kdr mutation (L1014F) and overexpressed cytochrome P450 enzymes [21].

### Experimental hut evaluation of Sovrenta® 15WP

#### Study site and experimental huts

The experimental hut trial was conducted at the CREC/LSHTM experimental hut station in Covè, Southern Benin (7.21’N, 2.34’E). The site is situated in a large rice-irrigated valley that supports the year-round production of *Anopheles gambiae* s.l. (constituting both *Anopheles coluzzii* and *An. gambiae* s.s.) [21]. The local vector population exhibits high levels of resistance to pyrethroids and organochlorines, with knockdown resistance (kdr) L1014F mutations present at ≥ 90% frequency and P450 enzyme overexpression contributing to over 200-fold increase in resistance intensity [21, 22]. Seven experimental huts of West African design were used and the trial lasted 12 months. The huts are constructed of concrete bricks with corrugated iron roofs. Inner walls and ceilings were plastered with either concrete or mud, prepared according to local construction practices. The huts are constructed of concrete bricks with corrugated iron roofs. Inner walls and ceilings were plastered with either concrete or mud, matching local construction practices. To prevent contamination from previous trials, all hut walls were replastered and allowed to cure for one month before the evaluation. Each hut was built on a concrete plinth surrounded by a water-filled moat to prevent the entry by scavenging ants. Mosquitoes entered through four window slits with a 1 cm window slits positioned on two sides of the hut. A wooden-framed veranda projected from the rear wall of each hut to collect exiting mosquitoes. Preliminary collections showed that the huts were equally attractive to Anopheline mosquitoes.

#### Experimental hut treatments

This hut trial evaluated seven treatments, each randomly assigned to one of seven experimental huts (Table 1). The efficacy of Sovrenta® 15WP was tested on both cement and mud substrates at a target dose of 120 mg a.i./m^2^, and compared to Actellic® 300CS—a WHO-prequalified, micro-encapsulated IRS formulation of pirimiphos-methyl applied at the recommended label rate of 1000 mg a.i./m^2^. Each Sovrenta® 15WP treatment (cement and mud) was applied in two replicate huts. An untreated cement-walled hut served as a negative control. IRS treatments were applied using calibrated Goizper® sprayers equipped with an 8002 flat-fan nozzle. Before spraying, hut walls and ceilings were measured, and spray swaths were marked with chalk to ensure precise application. Each swath was sprayed from top to bottom using a predetermined lance speed.

**Table 1:** Experimental hut treatments

S. No.	Treatment	Wall substrate	Hut replicate	IRS application dose (mg a.i./m^2^)
1	Control (Untreated hut)	Cement	N/A	N/A
2	Actellic® 300CS	Cement	N/A	1000
3	Actellic® 300CS	Mud	N/A	1000
4	SOVRENTA® 15WP	Cement	Replicate 1	120
5	SOVRENTA® 15WP	Cement	Replicate 2	120
6	SOVRENTA® 15WP	Mud	Replicate 1	120
7	SOVRENTA® 15WP	Mud	Replicate 2	120

#### Quality control of IRS applications

Before spraying, 5 filter papers (Whatman No.1) measuring 5 cm × 5 cm were fixed at 5 positions on the hut walls to be sprayed [19]. After spraying, they were removed, left to dry for 1 h and then wrapped in aluminium foil and stored at 4 °C (± 2 °C) in a refrigerator, after which they were shipped to Syngenta Crop Protection AG for chemical analysis to assess the quality of the spray applications. Spray quality was also assessed by measuring the volume of insecticide solution in the spray tank before and after spraying each hut. This allowed calculation of the amount of solution applied per hut and any deviation from the predetermined target volume.

#### Mosquito collections and processing

Trained human volunteers slept in the treated huts each night from dusk to dawn (21:00–6:00) for 6 nights per week during the entire study period to attract wild, free-flying mosquitoes into the experimental huts. Every morning, mosquitoes were collected from the different hut compartments (room, veranda) and placed into labelled plastic cups. They were then transferred to the field laboratory for morphological identification. Female *An. gambiae* s.l. specimens were separated and assessed for immediate mortality and blood-feeding status. Survivors were given access to a 10% (w/v) glucose solution, and their delayed mortality was monitored every 24 h for up to 168 h after collection across all treatments. Throughout the hut trial, sleepers were rotated daily between the experimental huts according to a pre-established Latin Square design to control for individual differences in attractiveness to mosquitoes. The efficacy of each treatment was assessed based on the following primary outcome measures:

- **Mortality:** the proportion of female mosquitoes that died every 24 h up to 168 h after collection.
- **Exophily:** the proportion of mosquitoes captured in the exit traps.
- **Blood-feeding rate:** the proportion of mosquitoes that had blood-fed.

#### Residual activity of insecticide treatments

The residual efficacy of the study treatments was assessed using in situ WHO wall cone bioassays [19], conducted 1 week after spraying and then monthly for 12 months. Fifty (50) unfed female mosquitoes (2–5 days old) of each strain (susceptible *An. gambiae* s.s. Kisumu, and pyrethroid-resistant *An. gambiae* s.l. Covè) were exposed to treated hut wall surfaces for 30 min. Mortality was then recorded every 24 h for up to 168 h post-exposure to evaluate the delayed mortality effect of Sovrenta® 15 WP.

#### Data management and analysis

During the experiments, observations were recorded on standardized raw data sheets designed for each study component (cone bioassays and experimental hut trials). Data were then double-entered into separate, pre-established Excel databases to ensure accuracy. Proportional outcomes from the hut trials (blood-feeding, exophily, and mortality) were analyzed using logistic regression in Stata version 18.1, with adjustments for repeated measures and the effects of sleepers, huts, and days of the trial. Mortality of wild *An. gambiae* s.l. was used to evaluate the non-inferiority of Sovrenta® 15 WP compared to Actellic® 300CS, employing a variable non-inferiority margin (NIM) as specified in recent WHO guidelines [23, 24]. Sovrenta® 15 WP was considered non-inferior to Actellic® 300CS if the lower bound of the 95% confidence interval of the odds ratio for mortality exceeded the NIM. Non-inferiority was assessed separately for each wall substrate and also combined across substrates. For the cone bioassays, mortality data were pooled for each treatment and substrate at each time point and compared against the WHO-recommended 80% mortality threshold [19].

To reinforce confidence in the robustness of the experimental hut results, a post-hoc simulation-based power analysis was performed using the ‘power_calculator_IRS’ function in R version 4.4.2. This analysis estimated the statistical power to detect non-inferiority of Sovrenta® 15WP applied at a target dose of 120 mg a.i./m^2^ to Actellic® 300CS for the primary outcome of mosquito mortality after 168 h with pooled data combining all replicates in concrete- and mud-walled huts at the 12-month timepoint.

#### Ethical considerations

The study received ethical approval from the Ethics Review Committee of the Benin Ministry of Health (Decision No. 21CNERS/Dos-021 dated 20 March 2023). Human volunteer sleepers who participated in the hut trials provided informed consent prior to their participation. The trial procedures and consent forms were explained in their local language by an interpreter. A stand-by nurse was present throughout the study to monitor sleepers for any signs of fever or persistent headache. Any sleeper who tested positive for malaria was immediately withdrawn from the study and received prompt treatment in accordance with local public health guidelines.

## Results

### Characterisation of mosquito strains

**Fig. 1:**
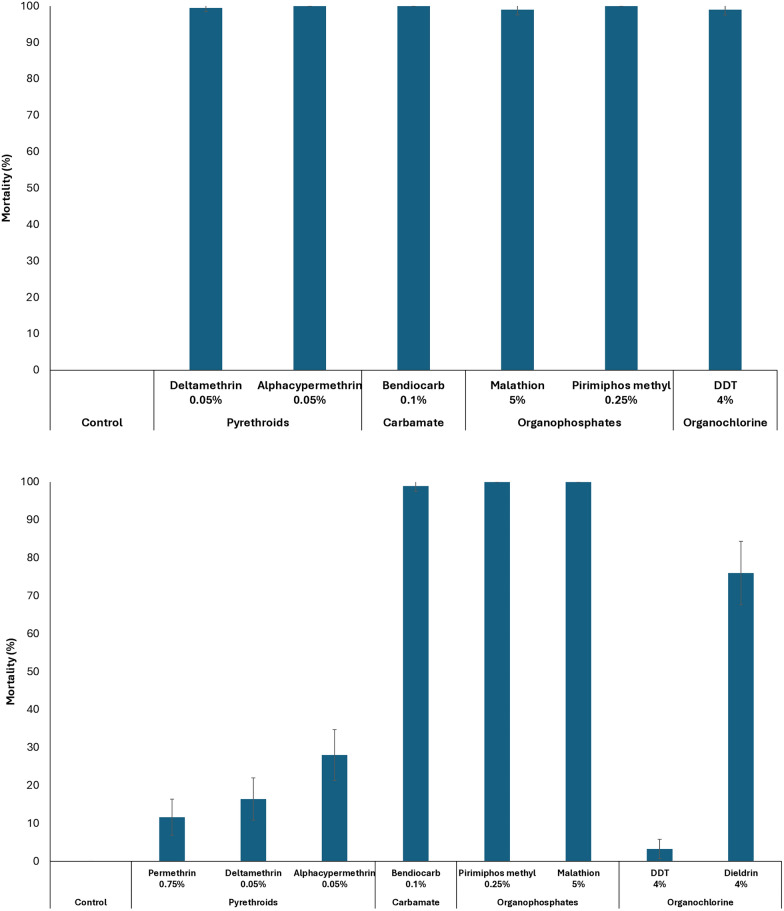
Results from routine WHO susceptibility tube tests conducted on *An. gambiae* Kisumu (**a**) and *An. gambiae* s.l. Covè (**b**) strains maintained at the CREC/LSHTM insectary before and during the study period. *Approximately 100 mosquitoes were tested per strain, exposed in batches of 25 to WHO tube tests using filter papers treated with the diagnostic concentration of each insecticide*

Routine susceptibility bioassays conducted during the study confirmed that the *An. gambiae* s.s. Kisumu strain was fully susceptible to pyrethroids, organophosphates, and carbamates, with bioassay mortality rates exceeding 98% (Fig. 1a). In contrast, the *An. gambiae* s.l. Covè population exhibited susceptibility to carbamates and organophosphates but showed resistance to pyrethroids, with mortality rates below 50% for permethrin (0.75%), deltamethrin (0.05%), and alphacypermethrin (0.05%) (Fig. 1b).

### Efficacy of Sovrenta® 15WP under laboratory conditions

**Fig. 2:**
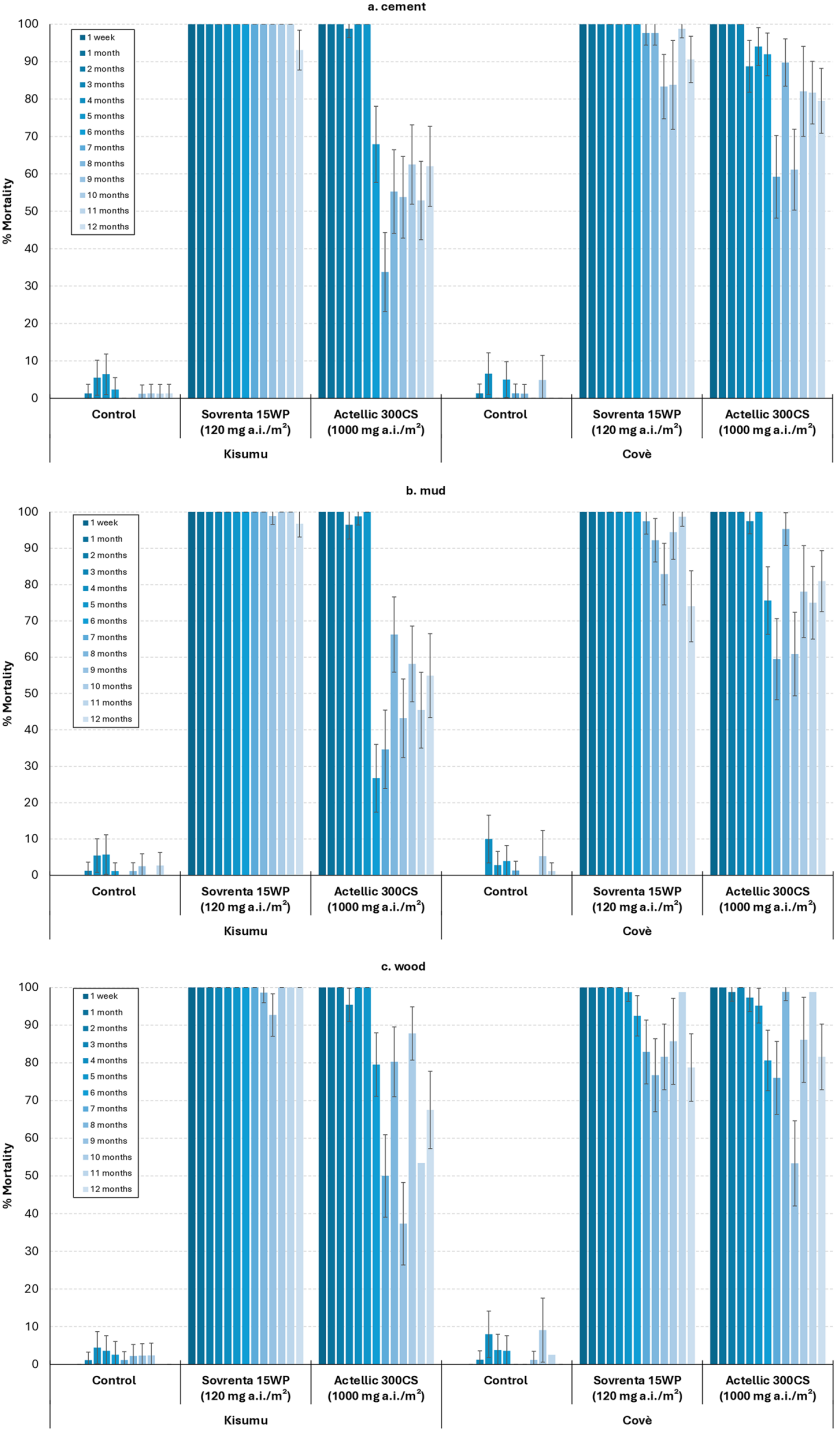
Mortality (%) at 168 h post-exposure of the susceptible *Anopheles gambiae* s.s. Kisumu strain and the pyrethroid-resistant *Anopheles gambiae* s.l. Covè strain following exposure to Sovrenta® 15WP (120 mg a.i./m^2^) and Actellic® 300CS (1000 mg a.i./m^2^) on cement (**a**), mud (**b**), and wood (**c**) blocks. *At each time point, approximately 80 mosquitoes were exposed to each treatment for 30 min. Error bars represent 95% confidence intervals*

Figure 2a–c show the overall mortality (168 h post-exposure) of the susceptible *An. gambiae* s.s. Kisumu and pyrethroid-resistant *An. gambiae* s.l. Covè strains in residual efficacy cone bioassays conducted in the laboratory on cement, mud, and wood substrates, respectively. Mosquito mortality with control untreated blocks remained below 5% for both strains at all time points, regardless of the substrate. On cement blocks, Sovrenta® 15WP 120 mg a.i./m^2^ maintained > 80% mortality for both the insecticide susceptible Kisumu strain and the pyrethroid-resistant Covè strain for all 12-months of testing (Fig. 2a). On mud blocks, Sovrenta® 15WP achieved > 80% mortality against the Kisumu strain for the full 12 months but dropped below 80% for the Covè strain after 11 months (Fig. 2b). On wood blocks, mortality exceeded 80% for the Kisumu strain throughout, while mortality for the Covè strain remained mostly above 80%, except during months 8 and 12 when it dipped slightly below 80% (Fig. 2c). In comparison, Actellic® 300CS showed less durable efficacy, with mortality generally falling below 80% after 6–7 months across all substrates.

## Experimental hut trial results

### Quality of IRS applications in experimental huts

**Fig. 3:**
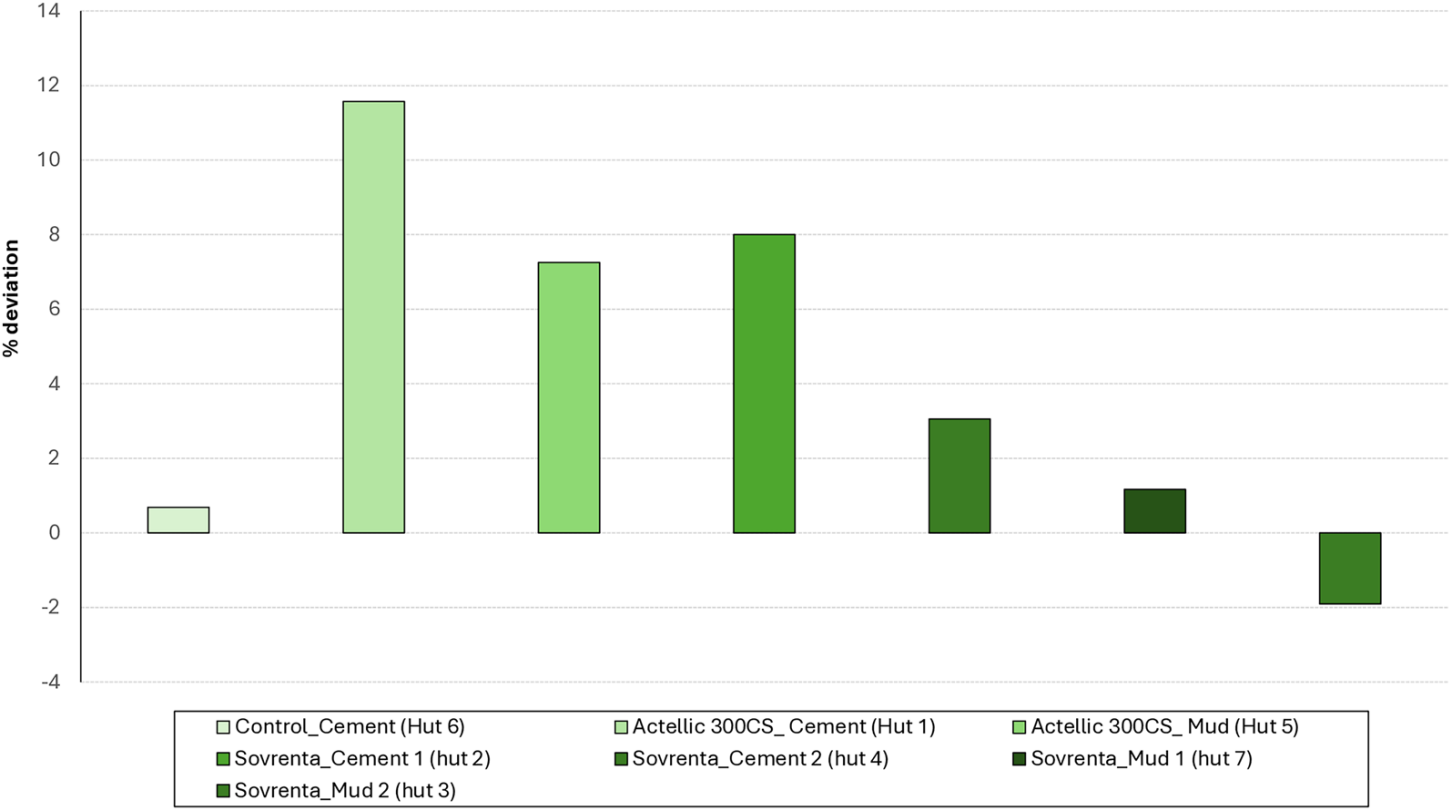
Percentage deviation from the target insecticide volume required for IRS applications in each hut treatment

IRS treatments were applied in April 2022. Six experimental huts received insecticide treatments, while the cement control hut was sprayed with water only. Sovrenta® 15WP was applied at a target dose of 120 mg a.i./m^2^ in two replicate huts cement and mud walled huts each, while Actellic® 300CS was applied at 1000 mg a.i./m^2^ in one cement-walled and one mud-walled hut. Figure 3 presents the percentage deviation from the required insecticide volume for each hut. Spray volumes ranged from -6% to + 12% deviation from the target, falling within the acceptable ± 30% range, indicating that treatments were correctly applied to the experimental huts.

### Entry and exiting rates of wild free-flying pyrethroid-resistant *An. gambiae s.l*

**Fig. 4:**
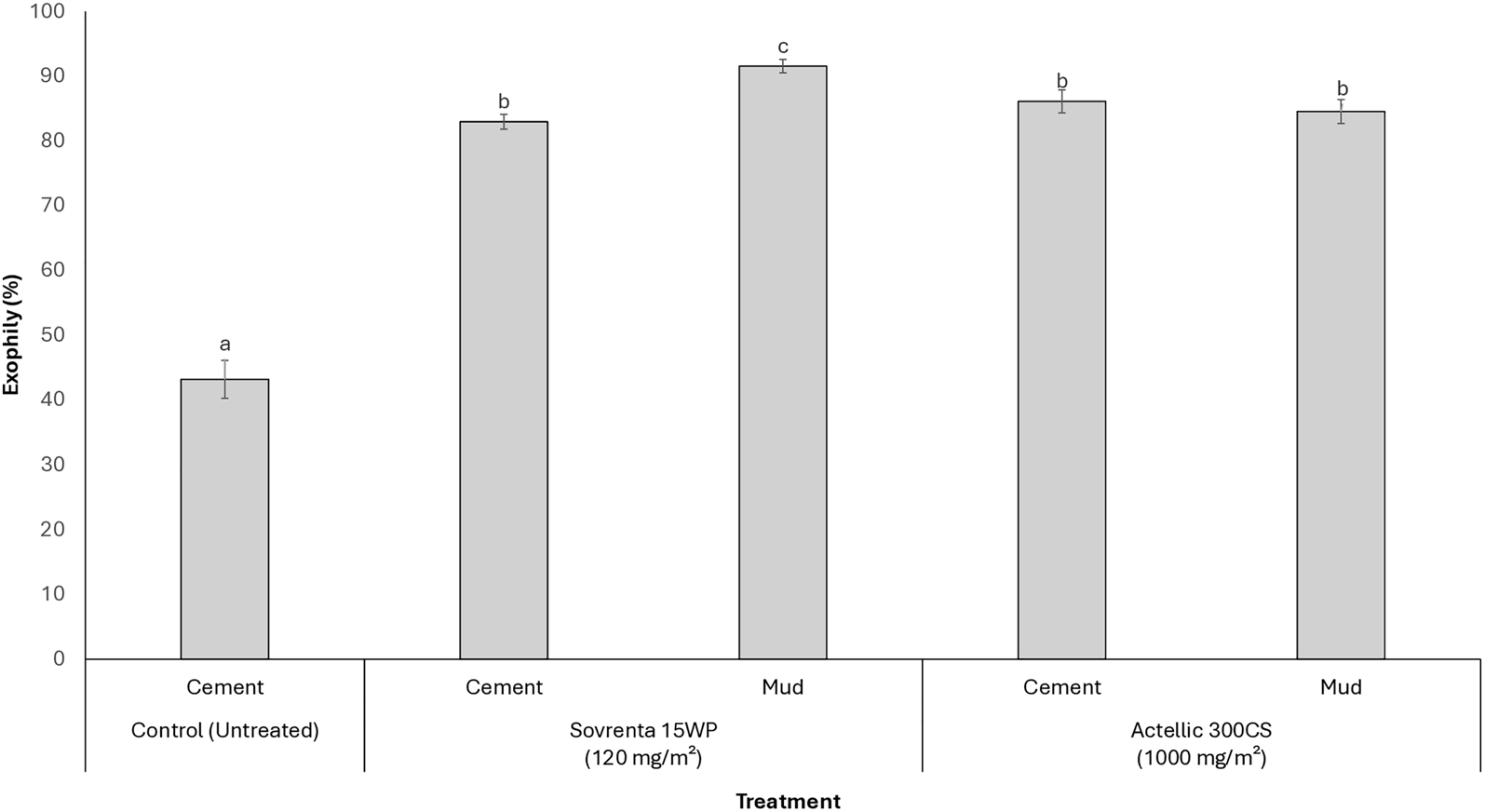
Exiting rates of wild, free-flying pyrethroid-resistant *An. gambiae* s.l. in experimental huts in Covè, Southern Benin. *Data for Sovrenta® 15WP on cement- and mud-walled huts are pooled across both replicates. Bars sharing the same letter label are not significantly different at the 5% level (p* > *0.05, logistic regression). Error bars represent 95% confidence intervals*

A summary of mosquito entry and exit patterns for wild, free-flying pyrethroid-resistant *An. gambiae* s.l. in the experimental huts is provided in Table 2. Figure 4 presents the overall exiting rates for each insecticide, combining data from replicate treatments. Across the study period, 12,850 female *An. gambiae* s.l. were collected, with the number of mosquitoes captured per hut treatment ranging from 1367 to 2532, corresponding to an average of 5 to 9 mosquitoes collected per hut per night. Like Actellic® 300CS, Sovrenta® 15WP demonstrated a deterrent effect compared to the untreated hut in both cement- and mud-walled huts. However, Sovrenta® 15WP exhibited a significantly stronger deterrent effect in mud-walled huts than in cement-walled huts (44–46% vs. 10–29%, *p* < 0.001). The exiting rate in the untreated cement-walled hut was 43%. All treated huts showed significantly higher mosquito exiting rates compared to the control hut (43% vs. 82–92%, *p* < 0.001). On cement-walled huts, Sovrenta® 15WP induced similar exophily as Actellic® 300CS (83% vs. 86%, *p* = 0.181). In contrast, on mud-walled huts, Sovrenta® 15WP produced significantly higher exophily than Actellic® 300CS (92% vs. 85%, *p* < 0.001).

**Table 2:** Entry and exiting rates of wild free-flying pyrethroid-resistant *An. gambiae* s.l. in experimental huts in Covè, Benin

Treatment	Control	Sovrenta® 15WP (120 mg a.i./m^2^)				Actellic® 300CS (1000 mg a.i./m^2^)	
Substrate	Cement	Cement 1	Cement 2	Mud 1	Mud 2	Cement	Mud
Total female collected	2532	2280	1805	1429	1367	1678	1759
Average per night	9	8	6	5	5	6	6
% deterrence	–	10	29	44	46	34	31
Total exiting	1093	1862	1527	1306	1254	1445	1487
% Exophily	43^a^	82^c^	85^b^	91^d^	92^d^	86^b^	85^b^
95% CI	41–45	80–83	83–86	90–93	90–93	84–88	83–86

### Mortality rates of wild pyrethroid-resistant *An. gambiae s.l.* Covè in experimental huts

**Fig. 5:**
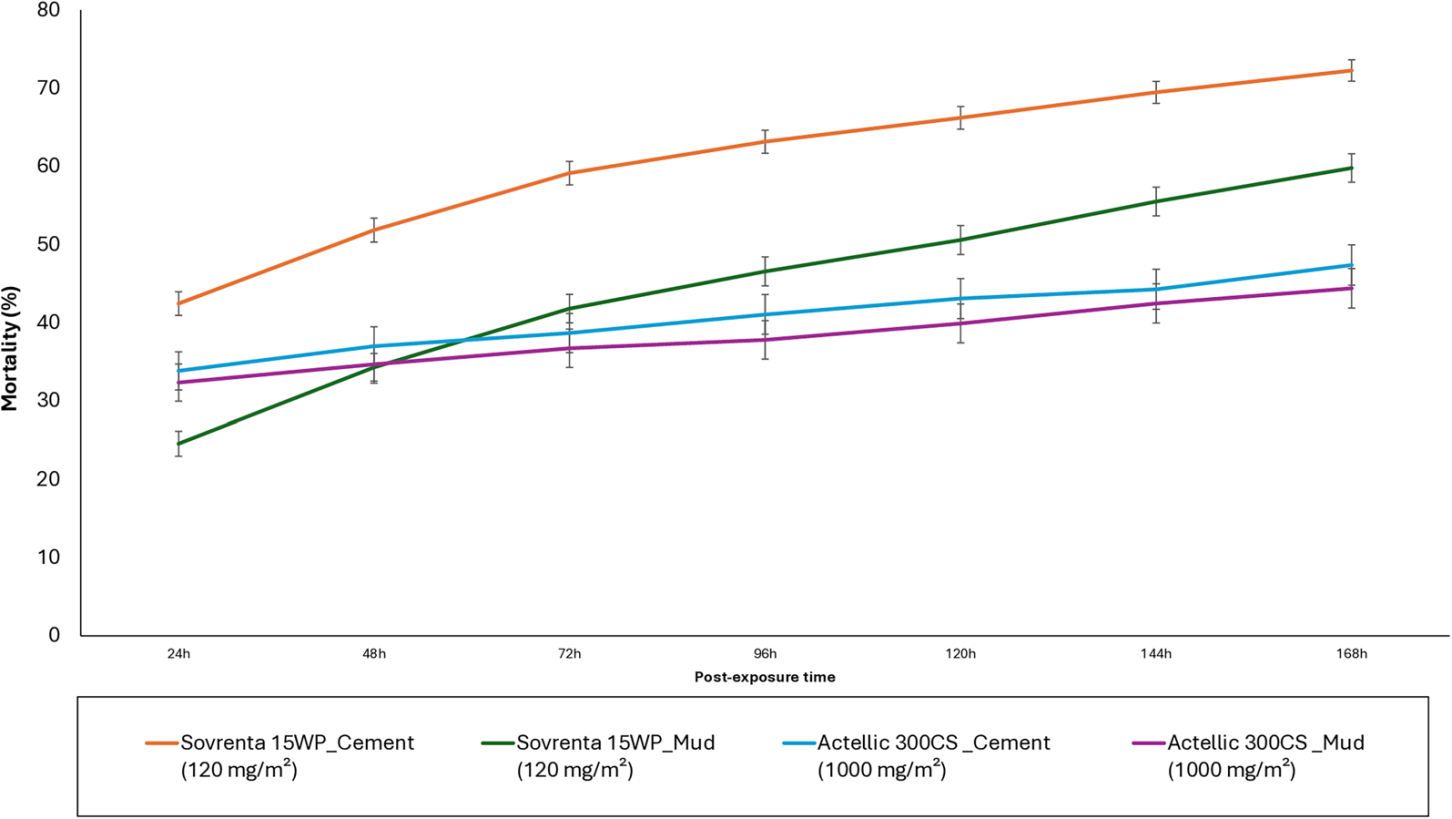
Overall mortality (24–168 h) of wild, free-flying, pyrethroid-resistant *An. gambiae* s.l. entering experimental huts in Covè, southern Benin. Overall mortality data for Sovrenta® 15WP cement and mud-walled huts are pooled for both replicates for 12 months of the study. For 168 h mortality, bars bearing the same letter label are not significantly different at the 5% level; *P* > 0.05, logistic regression. Error bars represent 95% Confidence Intervals. Sovrenta® 15WP induced a delayed mortality effect

To capture the delayed action of isocycloseram, the active ingredient in Sovrenta® 15WP, mortality of wild, free-flying, pyrethroid-resistant *An. gambiae* s.l. collected from experimental huts was recorded at 24-h intervals up to 168 h post-exposure for all treatments. Table 3 summarizes the overall mortality rates for each replicate hut treatment, while Fig. 5 presents the pooled mortality data by treatment. Mortality (168 h post-exposure) in the untreated cement-walled hut, remained very low (1%) throughout the 12-month trial (Table 3). Sovrenta® 15WP achieved an overall mortality of 59–72% at 168 h post-exposure, with significantly higher mortality in cement-walled huts compared to mud-walled huts (72% vs. 59–60%; *p* < 0.001). Over the 12 months study period, Actellic® 300CS also showed higher mortality in cement-walled huts than in mud-walled huts (47% vs. 44%; *p* = 0.001) (Table 3). Sovrenta® 15WP demonstrated a clear delayed mortality effect, regardless of wall substrate type (Fig. 5). Overall mortality increased from 42% at 24 h to 72% at 168 h on cement walls, and from 25 to 60% on mud walls. This pronounced delayed mortality effect was not observed with Actellic® 300CS, where mortality rose only modestly from 32–34% at 24 h to 44–47% at 168 h post-exposure.

**Table 3:** Summary of mortality (24 h and 168 h) results of wild, free-flying, pyrethroid-resistant *An. gambiae* s.l. entering experimental huts in Covè, southern Benin

Treatment		Control	Sovrenta® 15WP (120 mg a.i./m2)				Actellic® 300CS	
Substrate		Cement	Cement 1	Cement 2	Mud 1	Mud 2	Cement	Mud
Total female collected		2532	2280	1805	1429	1367	1678	1759
24 h post-exposure	Total dead	23	1016	718	359	328	568	569
	% Dead	1	45	40	25	24	34	32
	95% CI	0–2	43–47	38–42	23–27	22–26	32–36	30–35
168 h post-exposure	Total dead	23	1650	1302	862	810	795	781
	% Dead	1^a^	72^d^	72^d^	60^e^	59^e^	47^b^	44^c^
	95% CI	0–2	71–74	70–74	58–63	57–62	45–50	42–47

**Fig. 6:**
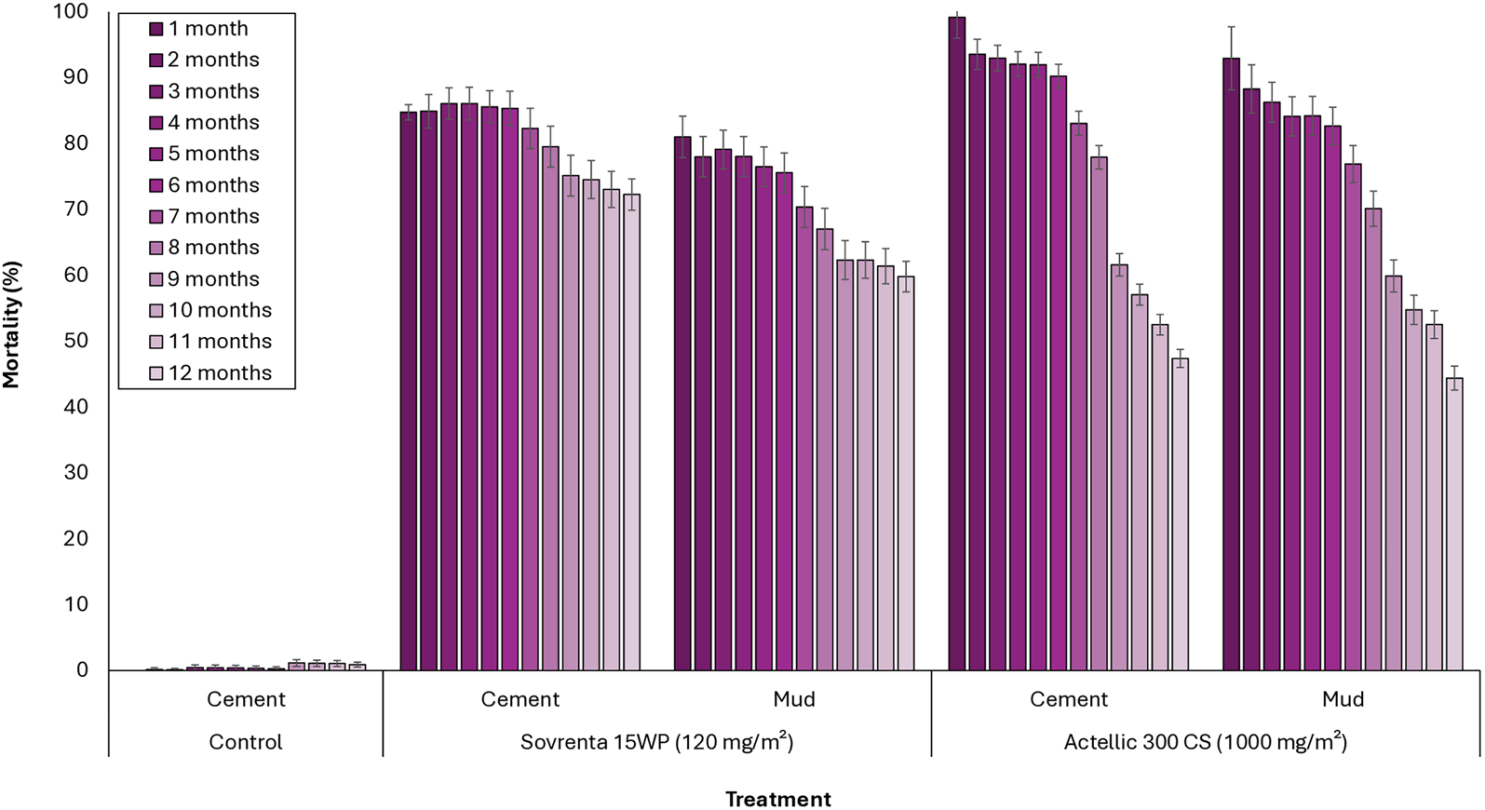
Monthly 168 h-mortality rates of wild free-flying pyrethroid-resistant *An. gambiae* s.l. entering experimental huts in Covè, Southern Benin for 12 months. Monthly mortality data for Sovrenta® 15WP cement and mud-walled huts are pooled for both replicates

Wild mosquito mortality with Sovrenta® 15WP ranged from 76 to 86% during the first six months and remained above 70% on cement-plastered huts and above 60% on mud-plastered huts throughout the 12-month study period (Fig. 6). In contrast, mortality with Actellic® 300CS ranged from 82 to 99% during the first six months but dropped below 60% in both cement- and mud-walled huts after nine months. Overall, these results demonstrate that Sovrenta® 15WP maintained a prolonged and consistent efficacy compared to Actellic® 300CS, sustaining effective control for up to 12 months on both wall substrate types.

### Blood feeding rates of wild pyrethroid-resistant *An. gambiae* s.l. Covè

**Fig. 7:**
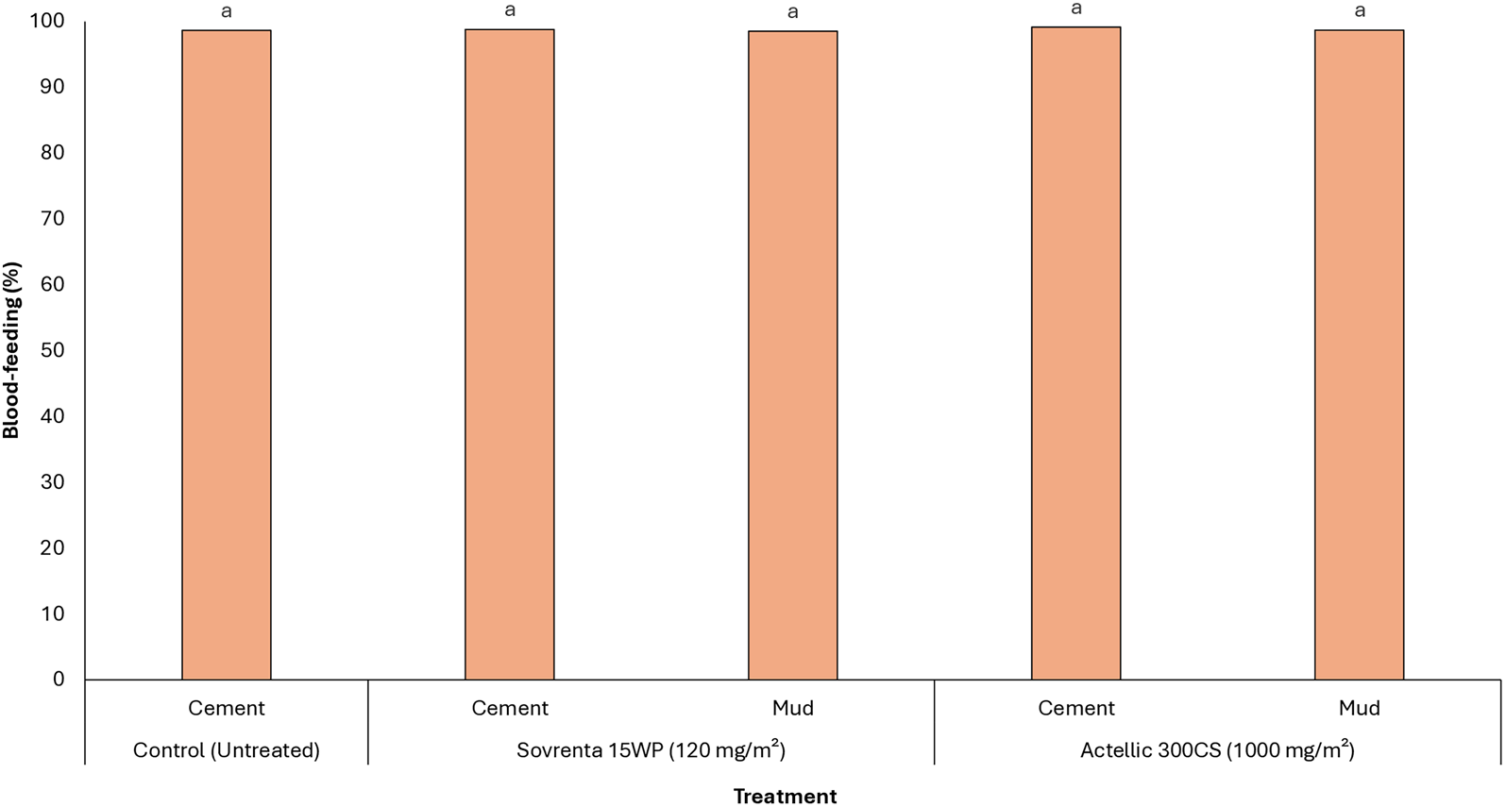
Blood-feeding rates of wild, free-flying, pyrethroid-resistant *An. gambiae* s.l. entering experimental huts in Covè, southern Benin. Blood-feeding data for Sovrenta® 15WP cement and mud-walled huts are pooled for both replicates. Bars bearing the same letter label are not significantly different at the 5% level

As expected with IRS treatments, blood-feeding rates remained consistently high across all treatments, exceeding 97% (Fig. 7). In the untreated cement-walled hut, mosquito blood-feeding was 99%. Pooled results for Sovrenta® 15WP in both cement- and mud-walled huts showed blood-feeding rates of 98%–99%, similar to the rates observed with Actellic® 300CS (99%, *p* > 0.05).

### Monthly residual cone bioassays on treated hut walls

**Fig. 8:**
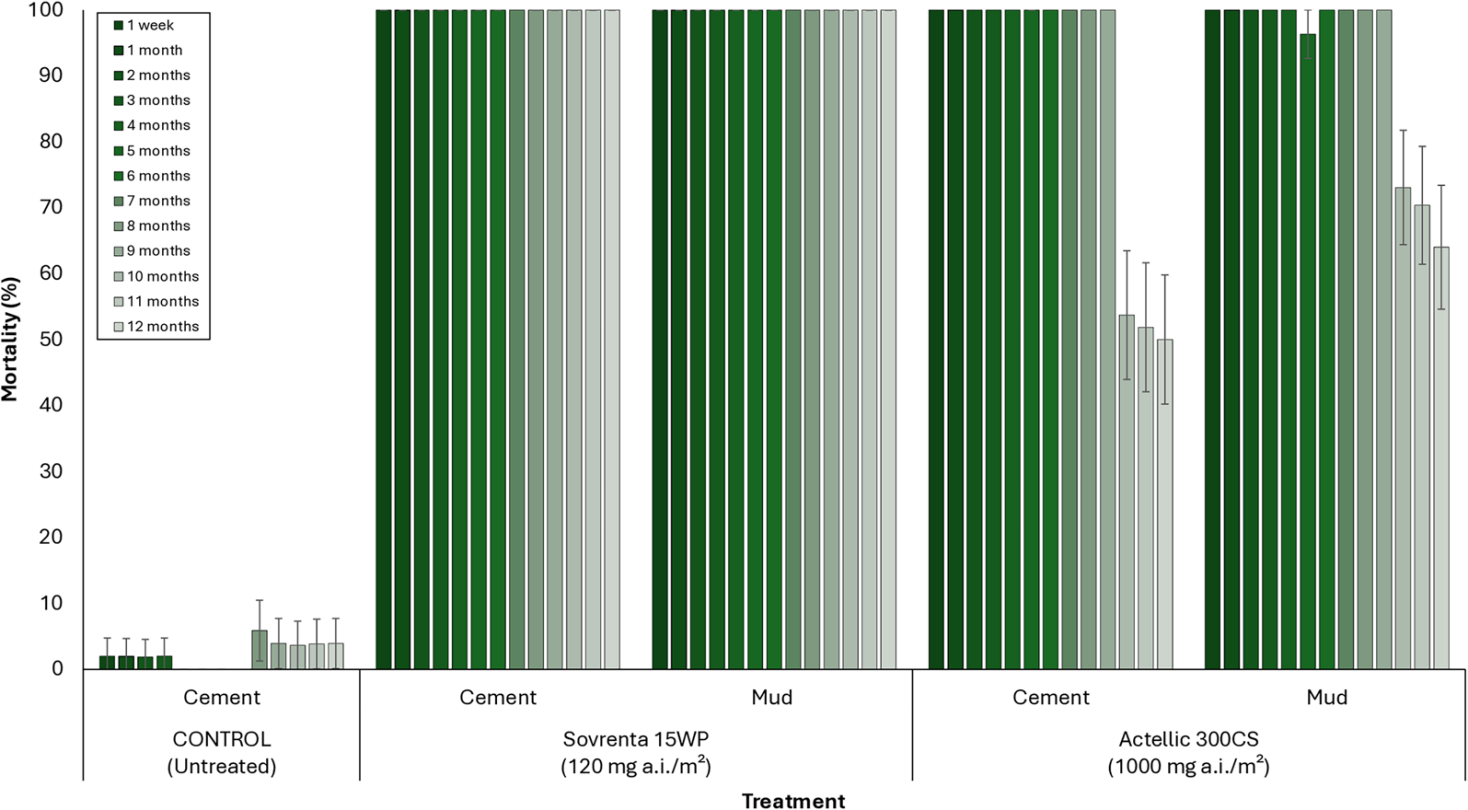
Monthly cone bioassays mortality rates of susceptible *An. gambiae* Kisumu on Sovrenta® 15WP treated walls. Sovrenta® 15WP treated cement and mud-walled huts is pooled for both replicate huts

**Fig. 9:**
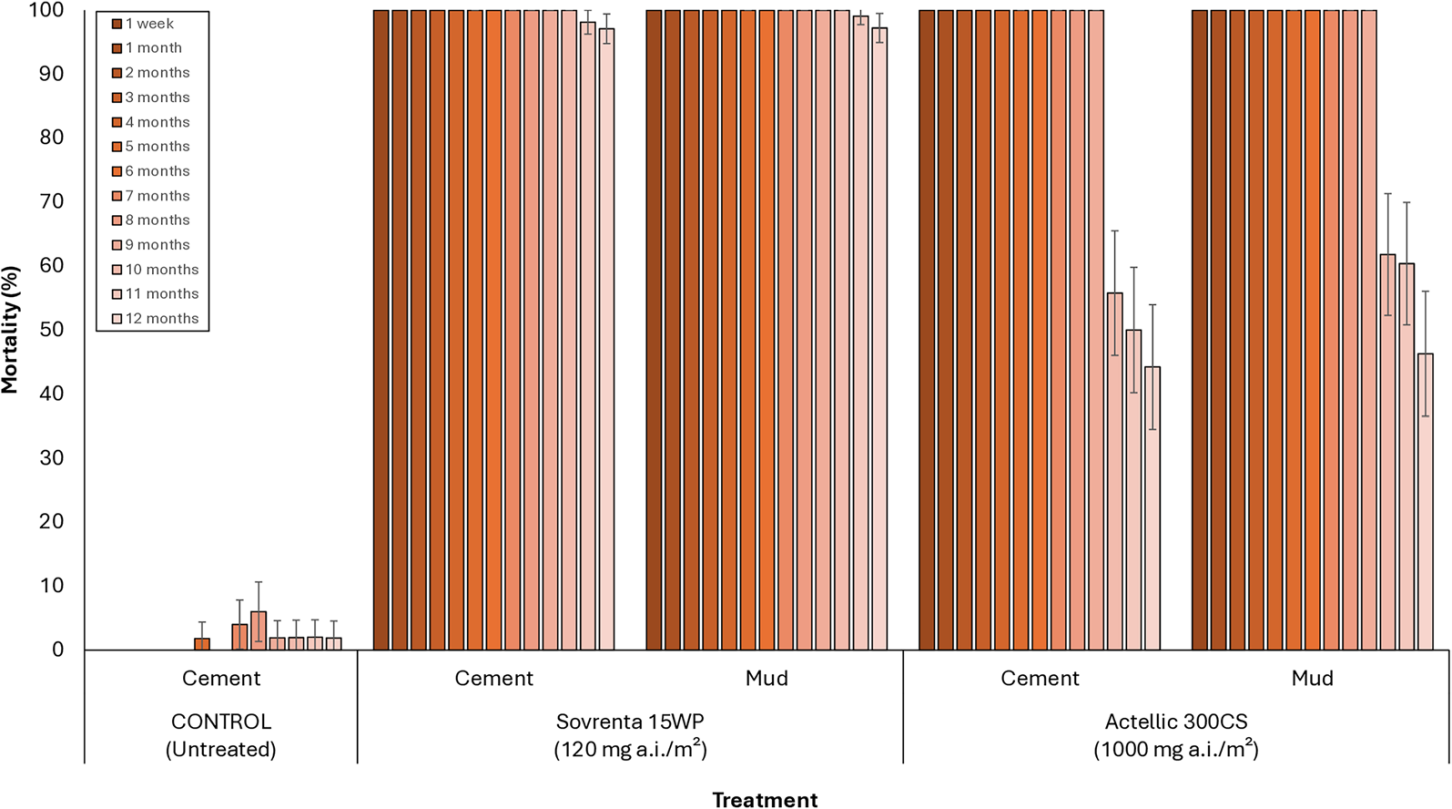
Monthly cone bioassays mortality rates of pyrethroid-resistant *An. gambiae* s.l. Cove on Sovrenta® 15WP treated walls. Sovrenta® 15WP treated cement and mud-walled huts is pooled for both replicate huts

Figures 8 and 9 present the mortality results (168 h post-exposure) from monthly in situ wall cone bioassays conducted over the 12-month trial for each hut treatment, using unfed susceptible *An. gambiae* s.s. (Kisumu) and pyrethroid-resistant *An. gambiae* s.l. (Covè) mosquitoes, respectively. In the untreated hut, mortality remained below 10% throughout the 12-month testing period for both strains. For the susceptible Kisumu strain, Sovrenta® 15WP demonstrated strong residual efficacy on both cement and mud substrates, consistently achieving mortality rates above 80% for the entire 12 months. Actellic® 300CS maintained > 80% mortality for 9 months on both cement and mud hut walls (Fig. 8). A similar trend was observed in WHO cone bioassays using the pyrethroid-resistant *An. gambiae* s.l. Covè strain. Sovrenta® 15WP consistently achieved > 80% mortality over the 12-month period on both cement and mud substrates, while Actellic® 300CS maintained > 80% mortality for 9 months on both wall types (Fig. 9).

### WHO non-inferiority analysis

Post-hoc power analysis demonstrated 100% power (95% CI: 99.3–100) to detect non-inferiority of Sovrenta® 15WP compared to Actellic® 300CS for the primary outcome of mosquito mortality at 168 h, using pooled data from both cement- and mud-walled huts after 12 months. Applying the variable odds ratio method recommended by WHO, the non-inferiority margins (NIMs) for mosquito mortality at 168 h over the 12-month trial were determined to be 0.70 for cement-walled huts, mud-walled huts, and when both wall types were combined (Table 4). The odds ratios comparing Sovrenta® 15WP to Actellic® 300CS were 3.38 (95% CI: 2.90–3.94) for cement-walled huts, 2.49 (95% CI: 2.10–2.95) for mud-walled huts, and 1.80 (95% CI: 1.53–2.11) when data from both wall types were combined. As the lower bounds of the 95% confidence intervals for these odds ratios exceeded the respective NIMs, Sovrenta® 15WP was found to be non-inferior to Actellic® 300CS against free-flying pyrethroid-resistant *An. gambiae* s.l. in both cement- and mud-walled experimental huts in Covè, Benin. Furthermore, Sovrenta® 15WP achieved significantly higher mortality rates than Actellic® 300CS (60–72% vs. 44–46%; *p* < 0.001), demonstrating not only non-inferiority but also statistical superiority over the 12-month trial.

**Table 4:** Non-inferiority of Sovrenta® 15 WP *vs* Actellic® 300CS for mortality of pyrethroid-resistant *An. gambiae s.l.* in experimental huts in Covè, Benin

Active comparator	Candidate	Substrate	NIM	OR	95% CIs	Target outcome	Test outcome
Actellic® 300CS (1000 mg a.i./m^2^)	Sovrenta® 15WP (120 mg a.i./m^2^)	Cement	0.70	3.38	2.90—3.94	Non-inferior	Superior
Actellic® 300CS (1000 mg a.i./m^2^)	Sovrenta® 15WP (120 mg a.i./m^2^)	Mud	0.70	2.49	2.10—2.95	Non-inferior	Superior
Actellic® 300CS (1000 mg a.i./m^2^)	Sovrenta® 15WP (120 mg a.i./m^2^)	Cement + Mud	0.70	1.80	1.53—2.11	Non-inferior	Superior

## Discussion

Developing new insecticide modes of action that can effectively control malaria vector populations resistant to existing public health insecticides is critical to sustaining the progress made in reducing the malaria disease burden [25, 26]. In this study, we assessed the efficacy and residual activity of Sovrenta® 15WP, a wettable powder formulation of isocycloseram, (active ingredient trademarked as PLINAZOLIN® technology) when applied as an indoor residual spray in both laboratory bioassays and semi-field experimental hut trials. Our findings demonstrate that Sovrenta® 15WP achieved significantly improved control of pyrethroid-resistant malaria vectors compared to Actellic® 300CS across multiple wall substrates, including cement, mud, and wood. This enhanced performance was sustained for up to 12 months following IRS application, indicating that Sovrenta® 15WP could be a valuable addition to the arsenal of tools available for malaria vector control.

Given the logistical and financial challenges associated with implementing IRS campaigns, the development of novel IRS formulations capable of providing extended control of wild, insecticide-resistant malaria vector populations is highly desirable [27]. Such long-lasting IRS insecticides could be deployed in a single annual campaign, offering year-round protection in high-endemic areas characterized by perennial malaria transmission [28, 29]. In both laboratory bioassays and semi-field experimental hut studies, Sovrenta® 15WP demonstrated a longer residual activity compared to Actellic® 300CS, maintaining effective vector control for 11–12 months when applied on local cement, mud, and wood wall substrates. This prolonged efficacy suggests that Sovrenta® 15WP could be a cost-effective IRS option in many high-endemic settings in Africa where existing IRS insecticides have delivered suboptimal results [30].

Sovrenta® 15WP demonstrated a clear delayed mortality effect against wild malaria vectors in the experimental hut trial, with mortality extending up to seven days post-exposure. Pirimiphos-methyl, the active ingredient in Actellic® 300CS, inhibits acetylcholinesterase, leading to rapid overstimulation and death. In contrast, isocycloseram, the active ingredient in Sovrenta® 15WP, binds allosterically to the GABA-gated chloride channel, gradually disrupting chloride ion flow and resulting in progressive hyperexcitation of the mosquito nervous system [14]. This mechanism can lead to delayed mortality over several days, a pattern also reported in other invertebrates exposed to isocycloseram [31]. Although the implications of this delayed mortality on malaria transmission are not yet fully understood, recent modeling studies suggest that it could significantly reduce the transmission potential of insecticide-resistant mosquitoes, including the major vector *Anopheles gambiae* s.l. [32].

To be recommended for use in IRS under existing WHO policy and to be listed by the WHO PQT/VCP as a prequalified vector control product, new IRS insecticides must demonstrate non-inferiority to a WHO prequalified IRS product in semi-field experimental hut trials conducted in at least two different ecological settings [24, 33, 34]. In this study, Sovrenta® 15WP was evaluated against Actellic® 300CS, a longer-acting micro-encapsulated formulation of the organophosphate pirimiphos-methyl that has been successfully deployed for malaria control across a wide range of epidemiological settings in Africa [35–38]. Over the 12-month trial, Sovrenta® 15WP was superior to Actellic® 300CS in inducing mortality of wild, free-flying pyrethroid-resistant *An. gambiae* s.l. mosquitoes entering both mud- and cement-walled experimental huts in Covè, Benin. Consequently, Sovrenta® 15WP met the WHO criteria for non-inferiority in this study. Based on these findings, along with results from trials conducted in other settings in sub-Saharan Africa, Sovrenta® 15WP was recently added to the WHO list of prequalified IRS insecticides. This endorsement paves the way for large-scale deployment of Sovrenta® 15WP, offering a promising tool for sustained control of malaria vector populations that have developed resistance to existing public health insecticides.

Before large-scale deployment of IRS with Sovrenta® 15WP in malaria vector control strategies, it is important to implement measures to preserve the susceptibility of local malaria vector populations to isocycloseram and thereby extend the insecticide’s useful life. This includes developing validated methods and establishing suitable discriminating concentrations for monitoring susceptibility in wild mosquito populations to support informed decision-making by control programmes. While this study demonstrated the efficacy of Sovrenta® 15WP against an *Anopheles gambiae* population with high levels of resistance to pyrethroids and organochlorines, additional studies should investigate potential cross-resistance of isocycloseram with other public health insecticides in different malaria vector species. Such studies will be critical for ensuring that Sovrenta® 15WP remains an effective and sustainable tool in malaria vector control programmes.

## Conclusion

Sovrenta® 15WP a wettable powder formulation of the newly discovered isoxazoline insecticide isocycloseram applied for IRS at the target application rate of 120 mg a.i. /m^2^ on mud, cement and wood wall substrates, provided improved and residual vector control lasting up to 12 months. The insecticide induced a delayed mortality effect in wild vector mosquitoes lasting up to 7 days post-exposure. Sovrenta® 15WP presents an additional IRS option for improving the control of pyrethroid-resistant malaria vectors and managing insecticide resistance through the rotation of IRS insecticides.

## Data Availability

No datasets were generated or analysed during the current study.

## Abbreviations

IRS: Indoor residual spraying
WHO: World Health Organization
WP: Wettable powder
PQ: Prequalification team
AI: Active ingredient
GABA: Gamma amino butyric acid
GLP: Good Laboratory Practice
CREC: Centre de Recherche Entomologique de Cotonou
LSHTM: London School of Hygiene & Tropical Medicine
PAMVERC: Pan African Malaria Vector Research Consortium
AIRID: African Institute for Research in Infectious Diseases
